# Characterization of Neurophysiological and Behavioral Changes, MRI Brain Volumetry and 1H MRS in zQ175 Knock-In Mouse Model of Huntington's Disease

**DOI:** 10.1371/journal.pone.0050717

**Published:** 2012-12-20

**Authors:** Taneli Heikkinen, Kimmo Lehtimäki, Nina Vartiainen, Jukka Puoliväli, Susan J. Hendricks, Jack R. Glaser, Amyaouch Bradaia, Kristian Wadel, Chrystelle Touller, Outi Kontkanen, Juha M. Yrjänheikki, Bruno Buisson, David Howland, Vahri Beaumont, Ignacio Munoz-Sanjuan, Larry C. Park

**Affiliations:** 1 Charles River Discovery Research Services, Kuopio, Finland; 2 MBF Labs, Williston, Vermont, United States of America; 3 Neuroservice SARL, Aix-en-Provence, France; 4 CHDI Management/CHDI Foundation, Los Angeles, California, United States of America; Tokyo Medical and Dental University, Japan

## Abstract

Huntington's disease (HD) is an autosomal neurodegenerative disorder, characterized by severe behavioral, cognitive, and motor deficits. Since the discovery of the *huntingtin* gene (*HTT*) mutation that causes the disease, several mouse lines have been developed using different gene constructs of *Htt*. Recently, a new model, the zQ175 knock-in (KI) mouse, was developed (see description by Menalled et al, [1]) in an attempt to have the *Htt* gene in a context and causing a phenotype that more closely mimics HD in humans. Here we confirm the behavioral phenotypes reported by Menalled et al [1], and extend the characterization to include brain volumetry, striatal metabolite concentration, and early neurophysiological changes. The overall reproducibility of the behavioral phenotype across the two independent laboratories demonstrates the utility of this new model. Further, important features reminiscent of human HD pathology are observed in zQ175 mice: compared to wild-type neurons, electrophysiological recordings from acute brain slices reveal that medium spiny neurons from zQ175 mice display a progressive hyperexcitability; glutamatergic transmission in the striatum is severely attenuated; decreased striatal and cortical volumes from 3 and 4 months of age in homo- and heterozygous mice, respectively, with whole brain volumes only decreased in homozygotes. MR spectroscopy reveals decreased concentrations of N-acetylaspartate and increased concentrations of glutamine, taurine and creatine + phosphocreatine in the striatum of 12-month old homozygotes, the latter also measured in 12-month-old heterozygotes. Motor, behavioral, and cognitive deficits in homozygotes occur concurrently with the structural and metabolic changes observed. In sum, the zQ175 KI model has robust behavioral, electrophysiological, and histopathological features that may be valuable in both furthering our understanding of HD-like pathophyisology and the evaluation of potential therapeutic strategies to slow the progression of disease.

## Introduction

Huntington's disease (HD) is an autosomal neurodegenerative disorder characterized by motor, behavioral, cognitive and metabolic dysfunction, and caused by an expansion of a CAG trinucleotide repeat region located in exon 1 of the *huntingtin* gene (*HTT*) on chromosome 4 [2]. HD is manifested when 36 or more CAG repeats are present, with 36–39 CAGs being incompletely penetrant and over 39 CAGs fully penetrant [3]. CAG repeat number inversely correlates with the age at which onset of motor symptoms occurs [4], which is typically 35–45 years of age in patients with 40–55 CAG repeats; the juvenile form of HD develops in carriers of 55 or more CAG repeats [2], [5]. HD is typically fatal 15–20 years after clinical diagnosis at motor symptom onset [6]. Neuropathologically, features in HD include a profound loss of medium spiny neurons in the striatum, degeneration of specific cortical areas, ventricular enlargement, and decreased striatal and cortical volume [7], [8].

Existing genetically-modified HD mouse lines use different *huntingtin* (*Htt*) constructs and are of three types: transgenic fragment models expressing N-terminally truncated mutant *Htt* (m*Htt*) proteins, transgenic full-length m*Htt*, or knock-in (KI) m*Htt* alleles [for review, see 9]. The first transgenic HD mouse models expressed truncated fragments of m*Htt* [10]. Typically these mice, such as the R6/2 strains, have rapidly developing disease signs including decreased body weight, motor and cognitive deficits starting as early as 6–8 weeks of age, and early mortality [10]. Disease onset in the BAC or YAC transgenic mouse strains expressing full-length m*Htt* varies from 8 weeks to 4 months, depending on the strain and the number of the CAG repeats, and they display variable onset and severity of various signs depending on the strain [11]. KI mouse models (in which the expanded CAG repeat is inserted into the mouse *Htt*) developed to date typically show more subtle behavioral, histopathological, and molecular phenotypes compared to the transgenic models that overexpress m*Htt* [12], [13], rendering these models less suited to preclinical compound testing where life cycle and numbers of mice are critical. For example, the CAG140 mouse (a KI line with 140 CAG repeats; [14]) displays early onset behavioral deficits with striatal neuronal loss and decreased volumes later [14], [15] but the robustness of these deficits may not be sufficient for preclinical compound testing studies [for review see 12]. The zQ175 KI mouse line was derived from a spontaneous germline CAG expansion in *Htt* from the CAG 140 line and initially characterized by those authors [1]. In this paper, we sought to independently confirm the behavioral deficits reported in zQ175 KI mice carrying approximately 179 CAG repeats [1]. We also further extend the characterization to include electrophysiological, morphological, volumetric and metabolic endpoints using acute slice physiology, magnetic resonance imaging (MRI) and magnetic resonance spectroscopy (MRS) *in vivo* during disease progression. Our work highlights significant, robust, dose-dependent, progressive, and early-onset alterations in these endpoints, which may prove useful to further investigate biological pathways affected by m*Htt* and to evaluate pharmacological or genetic interventions to modify disease onset and progression.

## Materials and Methods

### Animals

Experiments were conducted at Charles River Discovery Research Services, Kuopio, Finland, except the immunohistochemical measurements which were performed at MBF Labs, Williston, VT, USA, and the brain slice physiology conducted at Neuroservice SARL, France. All animal experiments were conducted according to the National Institute of Health (NIH) guidelines for the care and use of laboratory animals, and approved by the State Provincial Office of Southern Finland. For behavioral experiments, 10 female heterozygous (HET), 9 female homozygous (HOMO) zQ175 mice and 10 female wild-type littermate controls (WT) were acquired from Psychogenics Inc. (Tarrytown, NY, USA). zQ175 mice, originating from the CAG 140 mice (from germline CAG expansion) were generated by Psychogenics Inc. Homozygous, heterozygous and wild-type mice were generated by crossing heterozygous zQ175 mice on a C57B/L6J background. Genotyping and CAG repeat count were determined by Laragen Inc. (Culver City, CA, USA) at 10–15 days of age by PCR of tail snips. The average CAG repeat length was 178.3 (range 173 to 182) in heterozygous mice and 180.7 (range 175 to 185) in the homozygous mice. All the mice were housed in groups of up to 5 per cage, in a temperature (22±1°C) and humidity (30–70%) controlled environment with a normal light-dark cycle (7:00–20:00). All mice were housed in cages (dimensions: length 35 cm×width 19 cm×height 13 cm) with clean bedding covering the ground changed as needed to provide animals with dry bedding. In addition, a red mouse igloo was placed in each cage to provide environmental enrichment and shelter. Food (Purina Lab Diet 5001) and water were available ad libitum to mice in home cages. Water spouts were fitted with extensions to allow easy access from floor level. Mouse body weight was recorded weekly.

### Behavioral Tests

The tests were conducted during the dark phase of the light-dark cycle (between 23:00–5:00).

#### Open Field Test

Open field test was performed at the age of 2 months, 4 months, 8 months and 12 months. The mice were tested at approximately 10–30 lux of red light. The mice were brought to the experimental room for at least 1 h acclimation to the experimental room conditions prior to testing. The open field behavior was measured by automated Open Field Activity System and the data analyzed by Activity Monitor software (Med Associates Inc., St Albans, VT, USA). The activity chambers (27.3 cm×27.3 cm×20.3 cm) were equipped with infrared beams. Mice were placed in the center of the chamber and their behavior was recorded for 30 min in 5-minute bins.

#### Rearing-Climbing Test

Rearing climbing test was performed at the age of 2 months, 4 months, 8 months and 12 months. Each animal was placed into custom made pencil holder with wire mesh walls (height 15 cm×diameter 10.5 cm), and was tested for 5 minutes. Each test session was videotaped and the behavior was analyzed later from the videotapes by an experimenter blinded to the groups [modified from 15].

#### Procedural Two-Choice Swim Test

The swim tank was (dimensions: length 70 cm×width 30 cm×height 30 cm) filled with water (25±1°C) no higher than 1 cm from the top of a hidden escape platform. A hidden cylindrical platform (diameter 6 cm, height ∼9 cm) was placed either at right or left end of the tank. During testing only red lights were used in the room. During acquisition of the task, each mouse was given 8 training trials per day to swim towards or away from the right end (counterbalanced across subjects) to escape onto the platform. On each trial, mice were released in the center of the tank facing the experimenter and allowed to swim for up to 60 seconds or until they found the platform. If the mouse chose first the end of the tank with the platform “correct choice” was recorded, if the end without the platform “incorrect choice” was recorded. All animals were trained for 5 consecutive days. In all trials, choice and latency were recorded. The test was first commenced at 10 months of age and again at 12 months of age. The platform location was randomly placed on either the left or right side for each mouse and the platform location was the same throughout the testing. Each animal had the platform on the same end of the tank at both 10 and 12 months of age during testing.

### Electrophysiology

#### Acute striatal slice preparation

250 µm thick horizontal corticostriatal brain slices from mixed gender 2 – 9 month old zQ175 wild-type, heterozygous and homozygous mice were acutely prepared on a VT1200S vibrotome (Leica) in ice-cold sucrose solution composed of (in mM): sucrose 248, KCl 2, MgSO_4_ 2, NaH_2_PO_4_ 1.25, NaHCO_3_ 26, D-glucose 11.1, kynurenic acid 1. Slices containing the dorsal striatum were transferred into an incubation chamber containing artificial cerebrospinal fluid (ACSF) with a composition of (in mM): NaCl 125, KCl 2.5, CaCl_2_ 2, MgCl_2_ 1, NaHCO_3_ 25, NaH_2_PO4 1.25, D-glucose 25 at room temperature (RT) for at least 30 min to recover. All solutions were continuously bubbled with carbogen gas (95% CO2, 5% O2). For recordings, the slices were transferred into a 1 ml volume chamber and continuously superfused with ACSF (∼1.5 ml/min) at RT.

#### Patch-clamp recordings

Medium spiny neurons (MSNs) within the slice were visualized using an upright microscope equipped with infrared differential interference contrast (IR-DIC) and a 60× water-immersion objective (all from Olympus). Cells had an average membrane capacitance of 57.8±0.7 pF, n = 115, with no significant differences between genotypes or age groups noted. Recordings with an unstable or poor access resistance (R_a_) (>10% change over the period of recording, or initial R_a_ larger than 20 MΩ), were discarded. Average R_a_ was 9.5±0.28 MΩ, (n = 115), no significant difference between genotypes or age groups was noted.

Whole-cell patch clamp experiments were performed in both voltage-clamp and current clamp mode using a software-controlled MultiClamp 700B amplifier in combination with a Digidata 1440A digitizer (Molecular Devices). Patch pipettes were pulled from borosilicate glass capillaries (electrode resistance = 2–4 MΩ). For these experiments the intracellular solution contained (in mM): K-gluconate 105, KCl 30, MgCl_2_ 4, EGTA 0.3, HEPES 10, Na_2_ATP 4, Na_3_GTP 0.3 and Na-phosphocreatine 10, pH 7.35. Recordings were low-pass filtered at 2 kHz before being sampled at 10 kHz using Clampex 10 (Molecular Devices). Under voltage clamp control, cells were held at −80 mV and membrane capacitance (C_m_) and membrane resistance (R_m_) were calculated in response to a −10 mV hyperpolarizing pulse using the Membrane Test function of Clampex. Resting membrane potential (RMP), rheobase (minimum current injection required to elicit an action potential) and resultant action potential amplitude of the neurons were evaluated by switching to current clamp mode, prior to returning to voltage clamp control. Cortical stimulation to evoke glutamatergic excitatory post synaptic currents (eEPSCs) in the striatal MSNs were performed via a custom-built bipolar tungsten electrode that was placed between layer V of the cortex and deeper cortical layers. In a given MSN recording, cortical stimulation at a range of intensities (0.2–20 V) was used to determine the strength of glutamatergic transmission. Stimulation was repeated 20 times at a frequency of 0.1 Hz for each intensity level and the resultant eEPSCs were averaged per neuron. To determine the paired-pulse ratio, two stimulating pulses with a specific inter stimulus interval (ISI) were applied to the cortex (at 2–3 times the intensity needed to evoke a minimal response). Each individual cell was stimulated 5 times at a frequency of 0.1 Hz. The paired-pulse ratio was then calculated based on the average of these responses by dividing the amplitude of the second postsynaptic current recorded from the MSN with the first. Three age groups at 3–4 months, 6–7 months and 8–9 months of age were evaluated for recordings of MSN membrane parameters and evoked corticostriatal transmission. Results showed no statistically significant differences between the 6–7 month and 8–9 month age group within any given genotype or parameter evaluated, and thus these data were pooled. Between 10–37 neurons arising from at least 4 mice (range 4–7 mice) for each genotype-age group were recorded.

A separate set of experiments was performed in 2 month (n = 4 mice/genotype) and 7–9 month old mice (n = 12 mice/wild-type; n = 15 mice for zQ175 heterozygous; n = 4 for zQ175 for zQ175 homozygous) to isolate miniature excitatory postsynaptic currents (mEPSCs). In this instance, the intracellular solution comprised (in mM): Cs-methanesulfonate 100, Na-methanesulfonate 10, QX314-Cl 5, TEA-Cl 10, HEPES 10, CaCl_2_ 1, EGTA 10, MgATP 5, Na_3_GTP 0.5, pH 7.2. All recordings were performed with the addition of tetrodotoxin (TTX, 0.5 µM) and picrotoxin (50 µM) to the ACSF. Recordings were made at −80 mV for 20 min after observing stable activity for 10 min. The experiments were terminated by application of 10 µM CNQX to confirm that the events recorded were entirely AMPA receptor mediated. 23 neurons/genotype were recorded in the 2 month age group, and between 10–35 neurons/genotype recorded in the 7–9 month age group.

Data were analyzed with Clampfit 10 (Molecular Devices), Prism 5 (GraphPad) and Igor Pro 6 (Wavemetrics). mEPSCs recordings were analyzed for amplitude and frequency using Minianalysis (Synaptosoft). After the automatic detection of events, each trace was manually checked for false positives and false negatives.

### *In Vivo* MRI Volumetry and MRS

MRS and MRI measurements were performed for all mice at the age of 3 months, 4 months, 8 months and 12 months in a horizontal 7T magnet with bore size 160 mm (Magnex Scientific Ltd., Oxford, UK) equipped with Magnex gradient set (max. gradient strength 400 mT/m, bore 100 mm) interfaced to a Varian DirectDrive console (Varian, Inc., Palo Alto, CA). Linear volume coil was used for transmission and surface phased array coil for receiving (Rapid Biomedical GmbH, Rimpar, Germany). Isoflurane-anesthetized mice were fixed to a head holder and positioned in the magnet bore in a standard orientation relative to gradient coils. Rectal temperature (TC-1000 Temperature Controller, CWE Inc., Ardmore, PA, USA) and respiration (ECG Trigger Unit, Rapid Biomedical GmbH) were monitored throughout the study and the body temperature was kept at 36–37°C. For determination of volume of brain, striatum and cortex T2-weighted multi-slice (17 continuous slices) images were acquired using fast spin-echo sequence with TR/TEeff = 3000/36 ms, matrix size of 256×128, FOV of 20×20 mm2, slice thickness of 0.7 mm and 8 averages.

1H-MRS data were collected using the same experimental setup. Voxel of 2.5×2.5×3 mm^3^ was placed in the striatum of the mouse based on T2-weighed images collected as described above. Automatic 3D gradient echo shimming was initially used to adjust B0 homogeneity in the voxel and further manually shimmed to average water line-widths of 10.8±0.8 Hz. The water signal was suppressed using variable power RF pulses with optimized relaxation delays (VAPOR) to obtain B1 and T1 insensitivity. A PRESS sequence (TE = 19 ms) combined with outer volume suppression (OVS) is used for the pre-localization. Three OVS blocks were used interleaved with water suppression pulses. Data were collected by averaging 512 excitations (frequency corrected 16×32 blocks) with TR of 4 s, number of points 3000 and spectral width 3 kHz. In addition a reference spectrum without water suppression is collected from the identical voxel using the same acquisition parameters. Peak concentrations for major metabolites (e.g., NAA, Cho, Tau and Glx) were analyzed using LCModel (Stephen Provencher Inc., Oakville, Canada) and results are given relative to water content in tissue.

### Stereology

Total of 3 female and 3 male mice per genotype per age (wild-type, zQ175 heterozygous or homozygous at 4.5 months and 10 months) were used for stereological analysis. The brains were perfusion-fixed, frozen and sectioned at 60 µm sections at 360 µm intervals (Neuroscience Associates, Knoxville, TN). One series of sections per animal was processed for NeuN immunohistochemistry using overnight incubation with Millipore antibody MAB377, lot LV1634819, dilution 1∶100 000. Secondary antibody (Vector horse anti-mouse BA-2001, lot V1014, dilution 1∶248) and Vectastain ABC-kit (Vector) were used before visualization by DAB.

The stained sections were shipped to MBF Labs for stereologic analysis. All analyses were performed using a Olympus BX51 modified light microscope (Olympus, Japan) with Plan 2× (numerical aperture [N.A.] = 0.05) objective for delineation, and UPlanApo oil 100× (N.A. = 1.35) objective for counting, motorized specimen stage for automated sampling (Ludl Electronics, Hawthorne, NY), CCD color video camera (Microfire, Goleta, CA) and Stereo Investigator stereology software (v10.0, MBF Bioscience, Williston, VT).

The number of NeuN-positive neurons was quantified in bilateral striatum (Caudate-putamen and Nucleus Accumbens) using the Optical Fractionator stereological method. Sampling parameters were optimized to return an average coefficient of error of 0.05 (Gunderson (m = 1), [16]). On average, 426 and 412 cells per animal were marked in the nucleus accumbens and caudate-putamen respectively. Neurons were counted when the top of the nucleus came into focus within the 30 µm×30 µm counting frame. The dissector height was either 10 or 11 µm. A 2 µm guard zone was applied at the top and bottom of the section. Dissectors were spaced at a distance of 300 µm in the x and y dimensions for the nucleus accumbens and 700 µm in the x and y dimensions in the caudate-putamen. Cross-sectional neuronal area was estimated on every third cell during implementation of the Optical Fractionator probe by marking the somal boundaries of the cell from a random point near the midpoint of the soma using the Isotropic Nucleator probe with 4 rays [17]. The striatum was identified on a mean of 10 sections (ssf = 1/6) and the volume was analyzed based on Cavalieri's principle using a point spacing of 250 µm.

### Statistical Analysis

The statistical analyses were performed using IBM SPSS Statistics 19 and StatsDirect software unless otherwise specified. Behavioral and MRI measures were analyzed with Linear Mixed Models, analyzing the interactions for genotype, age and genotype×age interaction. If significant genotype effect was found One-way ANOVA followed by Dunnett's post-hoc test (comparison to wild-type mice) was used for each time point separately. The MRS data were analyzed with One-way ANOVA for each time point and metabolite separately. Stereology data were analyzed using 2-way ANOVA. All values are presented as mean ± Standard Error of Mean (SEM), and differences are considered to be statistically significant at the p<0.05 level. For the electrophysiological recordings, statistical analysis was performed using Prism 5 (GraphPad) and Igor Pro 6 (Wavemetrics). One-way ANOVAs with post hoc Tukey's test was used to assess statistical significance of the membrane parameters Rm, Cm, RMP, rheobase and spike amplitude, mean mEPSC frequency and amplitude values. A Two-way ANOVA with Bonferroni multiple comparison test was used to assess statistical significance of evoked corticostriatal transmission and paired pulse ratios. For mEPSCs, statistical differences in genotype were additionally assessed using D'Agostino and Pearson omnibus normality test to test for Gaussian distribution, followed by one sample t-test, to test whether means were significantly different than WT mean values from the frequency distribution analysis.

## Results

### Body Weight

**Figure 1:**
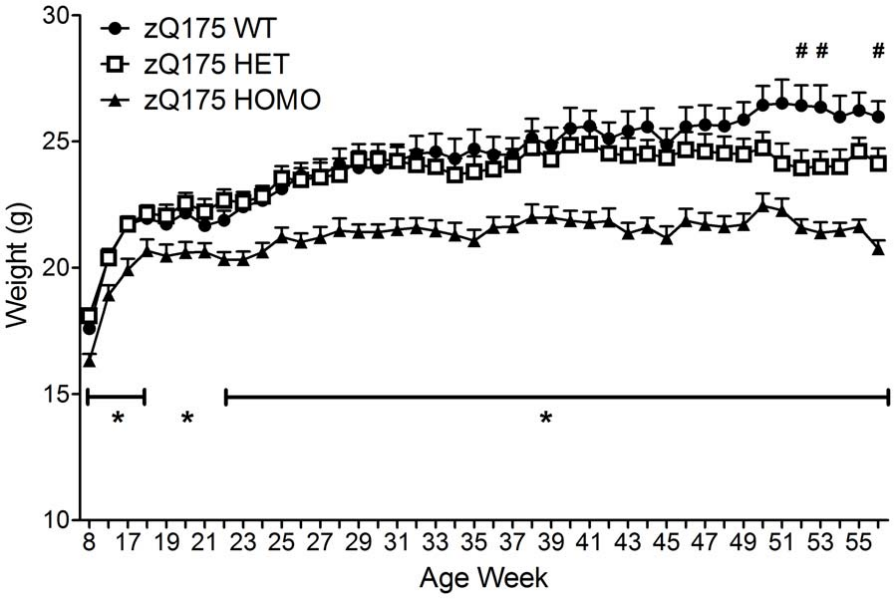
The body weight of the zQ175 and WT mice from 8 to 56 weeks of age. Data are presented as mean ± SEM. * p<0.05 zQ175 HOMO mice vs. zQ175 WT mice; # p<0.05 zQ175 HET mice vs. zQ175 WT mice (zQ175 WT, n = 10; zQ175 HET, n = 10; zQ175 HOMO, n = 9).

The homozygous zQ175 mice had decreased body weight on the first measurement point at 8 weeks of age compared to wild-type mice. The body weight of the homozygous mice remained significantly decreased until the end of the study, at 56 weeks of age, except on age weeks 19 and 21 compared to wild-type mice. The heterozygous mice had decreased body weight at 52, 53 and 56 weeks of age compared to wild-type mice (genotype main effect, F(2,838) = 359.0, p<0.001; age main effect, F(42,46) = 43.9, p<0.001; genotype×age interaction, F(84,46) = 1.6, p = 0.033; One-way ANOVA, p<0.05) (Fig. 1).

### Open Field

**Figure 2:**
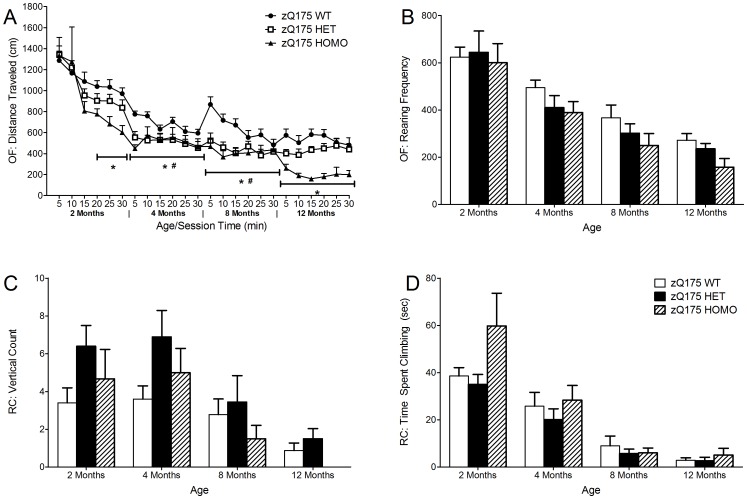
The open field motor test in zQ175 and WT mice. (A) Total distance traveled in the open field (OF) from 2 months to 12 months of age. (B) Total rearing frequency in the open field (OF) from 2 months to 12 months of age. (C) Rearing frequency in the rearing climbing test (RC) from 2 months to 12 months of age. (D) Time spent climbing in the rearing climbing test (RC) from 2 months to 12 months of age. Data are presented as mean ± SEM. * p<0.05 zQ175 HOMO mice vs. zQ175 WT mice; # p<0.05 zQ175 HET mice vs. zQ175 WT mice (zQ175 WT, n = 10; zQ175 HET, n = 10; zQ175 HOMO, n = 9).

The homozygous mice showed decreased horizontal activity compared to wild-type mice, i.e. they traveled shorter total distance already at 2 months of age, but only at the later phase of the test session, from 15 to 30 min. Furthermore, both the homozygous and heterozygous mice traveled a shorter total distance at 4 and 8 months of age, and the homozygous mice also at 12 months of age (genotype main effect, F(2,82) = 20.4, p<0.001; age main effect, F(3,42) = 65.0, p<0.001; One-way ANOVA, p<0.05) (Fig. 2A). Although there were no significant differences in the total rearing frequency between the genotypes there is a trend towards decreased rearing activity in the homozygous mice, namely at 12 months of age (genotype main effect, F(2,72) = 3.1, p = 0.052; age main effect, F(3,42) = 34.5, p<0.001) (Fig. 2B).

### Rearing Climbing

There was a significant main genotype effect in the rearing frequency in the rearing climbing test but no significant differences at separate time points were observed between the different genotypes (genotype main effect, F(2,79) = 4.6, p = 0.013; age main effect, F(3,51) = 22.6, p<0.001) (Fig. 2C). However, the rearing activity was increased in heterozygous mice at 2–4 months of age whereas homozygous mice displayed decreased rearing activity at 8 months but both of these trends failed to reach significance; at 12 months of age homozygous mice did not rear at all (Fig. 2C). There were no significant differences between the different genotypes in the time spent climbing (genotype main effect, F(2,57) = 2.9, p = 0.063; age main effect, F(3,35) = 34.2, p<0.001) (Fig. 2D). The climbing activity decreases in all the genotypes as the mice age from 2 to 12 months of age.

### Procedural Two-Choice Swim Test

**Figure 3:**
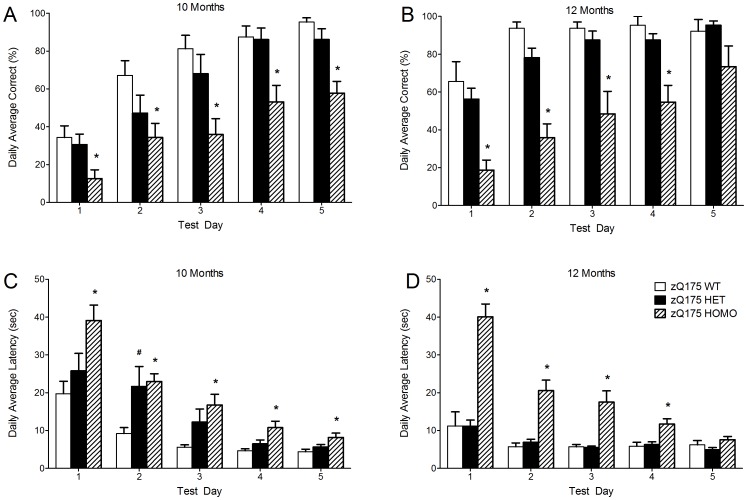
The procedural two-choice swim cognitive test in zQ175 and WT mice. (A–B) The percent correct choice in the procedural two-choice swim test at (A) 10 months and (B) 12 months of age. (C–D) The latency to find the platform in the procedural two-choice swim test at 10 months (C) and 12 months (D) of age. Data are presented as mean ± SEM. * p<0.05 zQ175 HOMO mice vs. zQ175 WT mice; # p<0.05 zQ175 HET mice vs. zQ175 WT mice (zQ175 WT, n = 10; zQ175 HET, n = 10; zQ175 HOMO, n = 9).

The procedural two-choice swim test was performed at 10 and 12 months of age. At 10 months of age the homozygous mice had a decreased percentage of correct choices on each test day (genotype main effect, F(2,94) = 30.0, p<0.001; test day main effect, F(4,49) = 44.6, p<0.001; One-way ANOVA, p<0.05) (Fig. 3A) and on test days 1–4 at 12 months of age compared to wild-type mice (genotype main effect, F(2,99) = 52.8, p<0.001; test day main effect, F(4,43) = 12.9, p<0.001; One-way ANOVA, p<0.05) (Fig. 3B). The heterozygous mice did not significantly differ from the wild-types (p>0.05) (Fig. 3B). Similarly, at 10 months of age the homozygous mice had increased latency on each test day and the heterozygous mice had an increased latency on test day 2 compared to wild-type mice (genotype main effect, F(2,67) = 18.1, p<0.001; test day main effect, F(4,30) = 29.2, p<0.001; One-way ANOVA, p<0.05) (Fig. 3C). At 12 months of age the homozygous mice had an increased latency on test days 1–4 compared to wild-type mice (genotype main effect, F(2,60) = 74.8, p<0.001; test day main effect, F(4,42) = 19.1, p<0.001; One-way ANOVA, p<0.05) whereas the heterozygous mice did not significantly differ from the wild-types (p>0.05) (Fig. 3D). Together these data show that the homozygous zQ175 mice were clearly impaired in this procedural memory test, requiring discriminative learning of stimulus – response association. This memory type is known to be dependent on striatal function [18].

### Electrophysiological Analysis

#### zQ175 striatal medium spiny neurons become progressively more excitable with age

**Figure 4:**
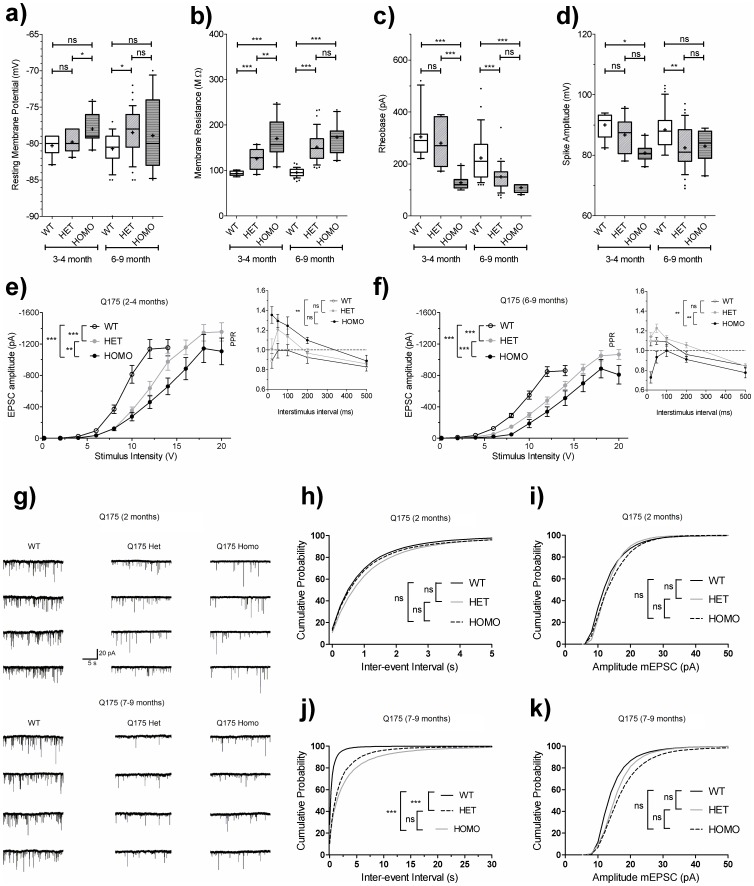
Electrophysiological deficits in zQ175 mice. Box and whisker plots of (A) resting membrane potential (B) membrane resistance (C) rheobase current and (D) action potential amplitude from MSNs in zQ175 wild-type, heterozygous and homozygous mice at 3–4 and 6–9 months of age. Box and whisker plots: + = mean, box = interquartile range, whiskers = 10–90 percentile, outliers = closed circles. (E–F) Measurement of evoked EPSCs in MSNs following cortical stimulation in zQ175 wild-type, heterozygous and homozygous mice at (E) 3–4 and (F) 6–9 months of age. (E–F inset) Paired-pulse ratio (inter-stimulus interval = 20–500 ms) at (E, inset) 3–4 months and (F, inset) 6–9 months for all genotypes. (G) Representative traces of mEPSCs from zQ175 wild-type, heterozygous and homozygous mice at 2 and 7–9 months of age. (H–K) Average cumulative plots of mEPSC inter-event interval (0.25 s bins; H, J) or amplitude (2 pA bins; i, k) in all 3 genotypes at (H–I) 2 months and (J–K) 7–9 months of age. zQ175 mice show a minor reduced frequency at 2 months of age, and a strongly depressed mEPSC frequency at 7–9 months of age (note difference in y axis in J compared to K). Mean mEPSC amplitude showed a slight increase in the frequency distribution towards higher amplitude events in the zQ175 mice, which appeared more pronounced in the 7–9 month age group (see also Table 1). Statistical analysis was performed as described in methods (one-way ANOVA values shown in H–K). ns = not significant, *p<0.05, **p<0.01, ***p<0.001.

Whole cell patch clamp recordings of medium spiny neuron (MSN) membrane properties were taken from acutely prepared brain slices from zQ175 wild-type, heterozygous and homozygous mice between 3 and 9 months of age, and were pooled into ‘early’ (3–4 months) and late (6–9 month) time-points for analysis (Fig. 4a–d). The resting membrane potential (RMP) of the MSNs were somewhat more depolarized in the zQ175 mice, although this was a modest and quite variable phenomenon of borderline significance (Fig. 4a). However, the zQ175 MSNs did show robust changes in membrane resistance (Rm) (Fig. 4b) and in rheobasic current (Fig. 4c), in line with these same observations previously made in MSNs from symptomatic R6/2 mice [19], [20] and from late stage TgCAG100 mice [20]. These results indicate that zQ175 MSNs are intrinsically more excitable than their wild-type counterparts, and that this progresses with age. In Q175 homozygous mice, the increase in Rm and decrease in rheobase was fully developed in the 3–4 month group, while in the zQ175 heterozygous mice; there was a progression of the phenotype between the earlier and later time-points evaluated. A trend to reduction in the resultant action potential amplitudes were also noted in the zQ175 mice at both ages, again of relatively variable statistical significance compared to wild-types (Fig. 4d), and in line with previous findings in the R6/2 mouse model [19].

#### zQ175 mice have impaired corticostriatal transmission and reduced glutamate release

Impaired corticostriatal connectivity and dysfunctional information processing in corticostriatal circuitry is a key feature of Huntington's disease [21]–[24] and dysfunction of corticostriatal transmission is emerging as a feature of many Huntington's disease mouse models [25]–[31]. To assess corticostriatal function in the zQ175 mice, glutamatergic excitatory post-synaptic currents (eEPSCs) were evoked in the slices by stimulating layer V cortical afferents innervating the striatum. In both 3–4 month (Fig. 4e) and 6–9 month (Fig. 4f) age groups, both zQ175 heterozygous and homozygous mice showed significant reduction in eEPSC amplitude for any given stimulus intensity applied relative to their age matched wild-type slices (n = 28–32 neurons per genotype).

To further attempt to identify the basis of this impairment in evoked glutamatergic transmission, a paired-pulse stimulation paradigm (20 ms–500 ms inter-stimulus interval) was employed to specifically assess alterations in glutamate release probability in the stimulated corticostriatal synapses [32]. In the 3–4 month age group, zQ175 homozygous mice displayed significantly higher paired-pulse ratios than those recorded from wild-type or heterozygous mice, which would be indicative of a decreased glutamate release probability from corticostriatal synapses in these mice (Fig. 4e, inset). However, in the later age group, this was reversed, with zQ175 homozygous corticostriatal synapses showing a greater tendency to paired pulse depression than either wild-type or heterozygous mice at short inter-stimulus intervals (Fig. 4f, inset). Heterozygous mice did not differ significantly from wild-type mice at either age group. It was thus difficult to confer with any confidence a particular locus of synaptic dysfunction (pre or postsynaptic) underlying the reduction in evoked corticostriatal transmission, using this particular assessment. We thus additionally measured spontaneous glutamate release onto MSNs in the presence of tetrodotoxin (TTX) to block action potential mediated release, and bicuculline to block any inhibitory transmission. In this scenario, the remaining miniature excitatory postsynaptic currents (mEPSCs; Fig. 4g) can arise from spontaneous release at both thalamostriatal and corticostriatal presynaptic terminals [33]. In 2 month old mice, the frequency (Fig. 4h) was somewhat reduced in zQ175 heterozygous or homozygous slices compared to wild type. While this was not significant (One-way ANOVA) when averaging the mean mEPSC frequency value obtained from each neuron recorded per group, analysis of the averaged frequency distribution curves (D'Agostino and Pearson omnibus normality test to test for Gaussian distribution, followed by one sample t-test) were highly significant (Table 1). The amplitude of mEPSCs (Fig. 4i) were also largely comparable at this age group, with if anything a small but significant increase in mean mEPSC amplitude noted in the zQ175 heterozygous an homozygous groups using the latter statistical test (Table 1). By 7–9 months of age, there was a further and quite profound reduction in mEPSC frequency in both the heterozygous and homozygous zQ175 mice, significant both when comparing mean mEPSC frequencies, and in the analysis of the frequency distribution curves (Fig. 4g, k; Table 1). A greater tendency for the appearance of higher amplitude mEPSCs in zQ175 heterozygous or homozygous mice compared to wild type was also noted, although this was again not significant when the mean amplitude measurements per neuron were compared per group (Table 1). Together, these data reflect a large and early reduction in glutamatergic innervation of the striatum, and shows that post-synaptic AMPA receptor function was not impaired in the zQ175 mice, and thus does not contribute to the impairment in evoked corticostriatal transmission observed.

**Table 1:** Electrophysiological analysis of MSNs from zQ175 and WT mice.

Genotype	Age (months)	Mean mESPC frequency (Hz)	Significance (P)		Mean mESPC Amplitude (pA)	Significance (P)		n
			ANOVA	D'A&P		ANOVA	D'A&P	
**zQ175 WT**	2	0.97±0.1	n/a	ns	14.5±0.7	n/a	ns	23
	7–9	1.63±0.18	n/a	ns	16.2±0.5	n/a	ns	34
**zQ175 HET**	2	0.84±0.1	ns	****	14.3±0.6	ns	****	23
	7–9	0.29±0.05	***	****	17.3±0.6	ns	****	35
**zQ175 HOM**	2	0.63±0.1	ns	****	15.7±0.9	ns	****	23
	7–9	0.35±0.06	***	****	17.9±1.2	ns	****	10

Mean mEPSC frequency and amplitude in zQ175 wild type (WT), heterozygous (HET) and homozygous (HOM) MSNs at 2 and 7–9 months of age. Statistical comparisons were made between aged matched WT and zQ175 HET and HOM mice using either a ONE-WAY ANOVA with Tukey's post-test (ANOVA) on mean frequency and amplitude values, or by D'Agostino and Pearson omnibus normality test followed by one sample t-test, to test whether means were significantly different than WT mean values from frequency distribution analysis (D'A & P). ns = not significant,

*p<0.05,

**p<0.01,

*** p<0.001,

**** p<0.0001.

### 1H MR Imaging and Spectroscopy

**Figure 5:**
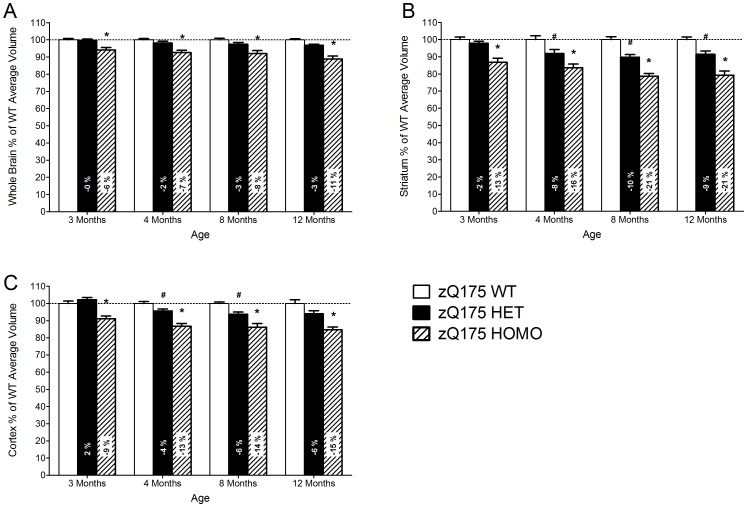
The percent changes of *in vivo* MRI brain volume in Q175 mice. (A) Whole brain, (B) striatal and (C) cortical volumes from 3 months to 12 months of age as measured by MRI. Data are presented as mean ± SEM normalized to WT control group at each time point. Data are presented as mean ± SEM. * p<0.05 zQ175 HOMO mice vs. zQ175 WT mice; # p<0.05 zQ175 HET mice vs. zQ175 WT mice (zQ175 WT, n = 10; zQ175 HET, n = 10; zQ175 HOMO, n = 9).

The homozygous zQ175 mice had decreased whole brain (genotype main effect, F(2,106) = 66.8, p<0.001; age main effect, F(4,39) = 3.3, p<0.001; One-way ANOVA, p<0.05), striatal (genotype main effect, F(2,105) = 107.8, p<0.001; age main effect, F(4,44) = 30.7, p<0.001; One-way ANOVA, p<0.05) and cortical volumes (genotype main effect, F(2,98) = 83.8, p<0.001; age main effect, F(4,37) = 6.2, p = 0.001; One-way ANOVA, p<0.05) at 3, 4, 8, and 12 months of age (Fig. 5A, B, C). The heterozygous zQ175 mice had decreased striatal volumes at 4, 8 and 12 months of age, and decreased cortical volumes at 4 and 8 months of age compared to wild-type mice (p<0.05) (Fig. 5A, B, C). Whole brain volumes were not significantly decreased in the heterozygous mice but there is a non-significant trend towards decreased volumes at 12 months of age (One-way ANOVA, Dunnett's post-hoc, p = 0.084) (Fig. 5A). For the longitudinal effect, the data is presented as normalized to wild-type control group at each time point.

Volumetric changes were most pronounced in the striatum (down to approximately −21% at 12 months) in homozygous mice, followed by cortical atrophy (down to approximately −15% at 12 months) and whole brain atrophy (down to approximately −11% at 12 months) (Fig. 5A, B, C). Heterozygous mice displayed similar brain volumetric changes, but the changes were less pronounced (Fig. 5A, B, C).

**Figure 6:**
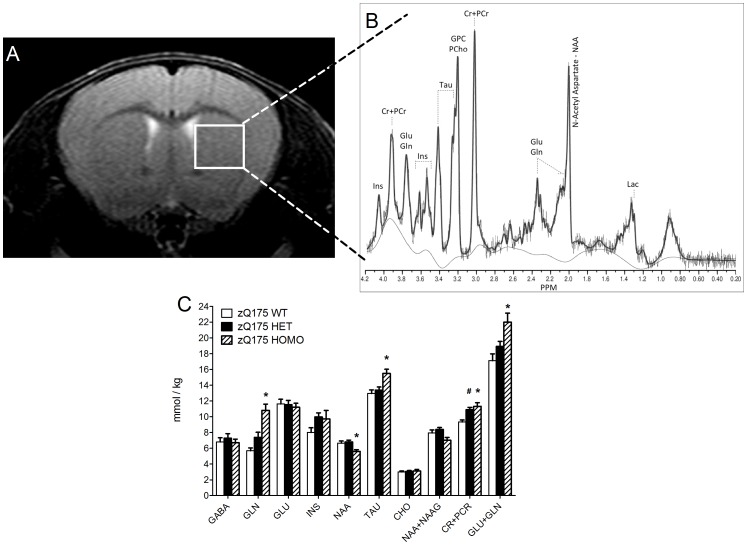
*In vivo* MRS in the striatum of Q175 and WT mice. (A) Representative T2-weighted MRI and localized volume from which the (B) MR spectra were recorded. (C) The concentrations of striatal metabolites at 12 months of age as measured by MR spectroscopy. Data are presented as mean SEM. Abbreviations: GABA, gamma-aminobutyric acid; GLN, glutamine; GLU, glutamate; INS, myo-inositol; NAA, N-acetylaspartate; TAU, taurine; CHO, choline; NAA+NAAG, N-acetylaspartate+N-acetylaspartylglutamate; CR+PCR, creatine+phosphocreatine; GLU+GLN, glutamate+glutamine. Data are presented as mean ± SEM. * p<0.05 zQ175 HOMO mice vs. zQ175 WT mice; # p<0.05 zQ175 HET mice vs. zQ175 WT mice (zQ175 WT, n = 10; zQ175 HET, n = 10; zQ175 HOMO, n = 9).

**Figure 7:**
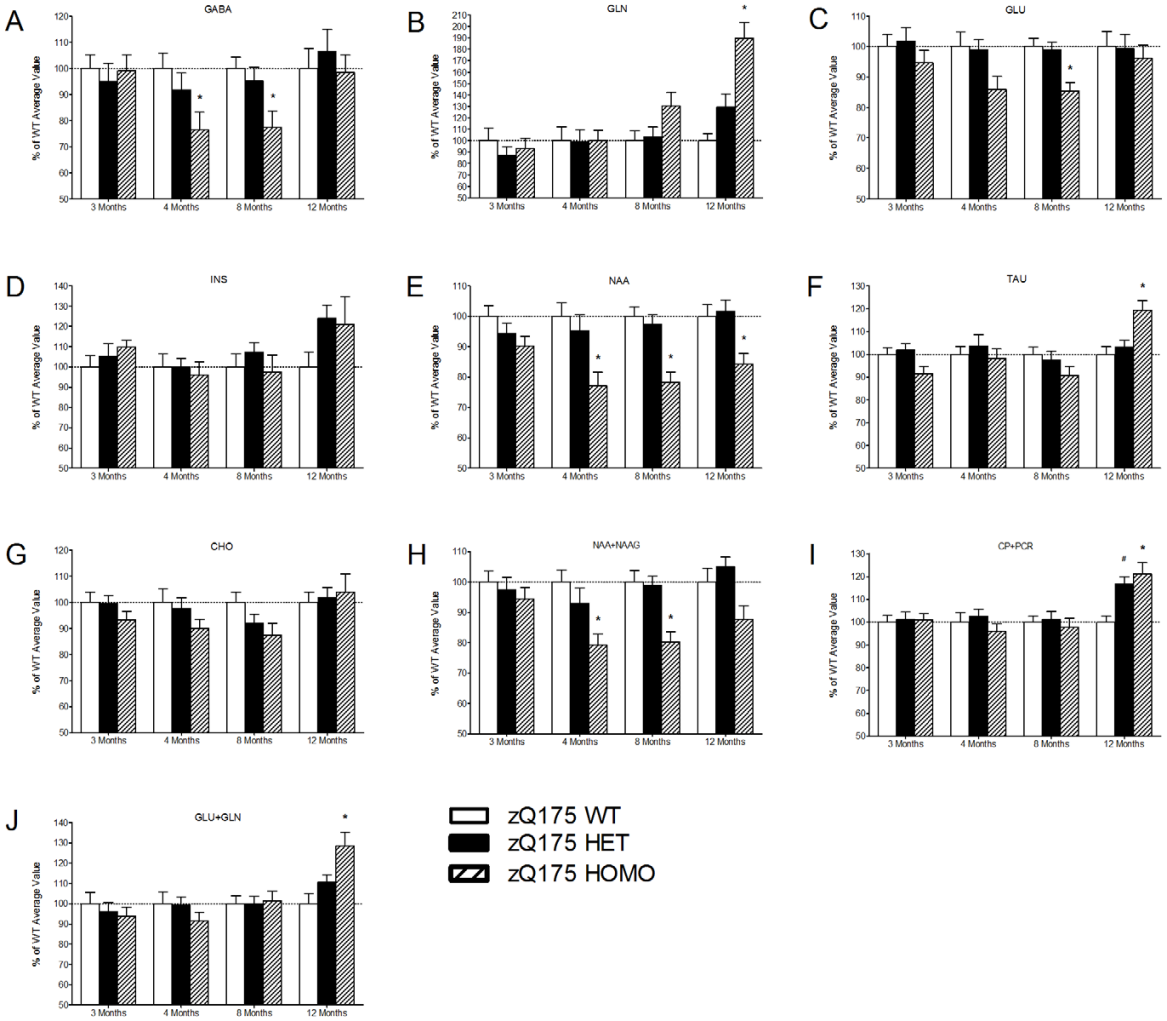
The percent changes of 1H MRS metabolites in the striatum of zQ175 mice. Longitudinal changes of individual 1H MRS observed metabolites in the striatum as given normalized to values obtained from WT control group at each time point. The striatal concentrations are presented for (A) GABA, gamma-aminobutyric acid, (B) GLN, glutamine, (C) GLU, glutamate, (D) INS, myo-inositol, (E) NAA, N-acetylaspartate, (F) TAU, taurine, (G) CHO, choline, (H) NAA+NAAG, N-acetylaspartate+N-acetylaspartylglutamate, (I) CR+PCR, creatine+phosphocreatine and (J) GLU+GLN, glutamate+glutamine. Data are presented as mean ± SEM. * p<0.05 zQ175 HOMO mice vs. zQ175 WT mice; # p<0.05 zQ175 HET mice vs. zQ175 WT mice (zQ175 WT, n = 10; zQ175 HET, n = 10; zQ175 HOMO, n = 9).

Various brain transmitter and metabolite changes are also observed in the striatum of zQ175 mice over time (Fig. 6 and 7). For the longitudinal effect between the genotypes over the observation period from 3 months up to 12 months, individual metabolites are shown in Figure 6 with values normalized to wild-type control group at each time point. No significant differences between the groups were found in any of the observed metabolites at 3 months of age (One-way ANOVA, p>0.05) (Fig. 7). At both 4 and 8 months of age, the homozygous mice had decreased concentrations of GABA (approximately −23%), glutamate (approximately −14%), N-acetylaspartate (NAA; approximately −22%) and NAA+NAAG (approximately −20%) compared to wild-type mice (One-way ANOVA, Dunnett's post-hoc, p<0.05) (Fig. 7). At 8 months of age the homozygous mice had decreased concentrations of glutamate (approximately −14%) (One-way ANOVA, Dunnett's post-hoc, p<0.05) (Fig. 7). At 12 months of age the homozygous mice had decreased concentrations of N-acetylaspartate and increased concentrations of glutamine, taurine and creatine+phosphocreatine as well as glutamine+glutamate, and the heterozygous mice had increased concentrations of creatine+phosphocreatine (approximately +17%) (One-way ANOVA, Dunnett's post-hoc, p<0.05) (Fig. 6, 7). Interestingly, apparent recovery of GABA and glutamate levels in homozygous mice is seen from 8 to 12 months (Fig. 6, 7). Although the increasing metabolite levels are often detected for many of the metabolites in HD, e.g. myo-inositol, cholines, glutamine, taurine and total creatines, similar increasing pattern from 8 to 12 months is systematically observed for all of the metabolites in our data for both the heterozygous and homozygous groups (Fig. 7). Absolute values, presented in this study, are derived by using the water signal acquired from the same voxel as an internal reference which in turn creates additional variable that must be taken into account. Systematically increased absolute values may result from either global increase of metabolic products or from decreased NMR visible water content.

A power analysis of the MRI volumetry data at 12 months of age (StatsDirect statistical software, UK) indicated that a group size of 8–12 mice per genotype would be sufficient to detect a 50% improvement in whole brain, striatal and cortical volumetric loss in homozygous zQ175 mice (α = 0.05, 80% power, see table S1 for details). For heterozygous zQ75, a group size of 24–26 mice per genotype would be needed for a 50% improvement in whole brain and striatum volume, and 44 mice for cortical volume. Similarly, for a selection of MRS metabolites at 12 months of age, 26–29 mice per genotype would be needed to detect a 50% recovery of GLN, NAA and CR+PCR level in homozygous zQ175 mice; whereas, at least 78 mice per genotype are needed for GLN, and 21 for CR+PCR in heterozygous zQ175 (Table S1).

### Stereology

The striatum, comprised of the Caudate Putamen (CPu) and Nucleus Accumbens (NA), was analyzed for changes in regional volume, neuron number and neuronal cross-sectional area due to genotype, age and interactions of genotype and age, using stereology (Table 2).

**Table 2:** Immunohistological analysis in brains of zQ175 and WT mice.

	Neuronal number (bilateral)	Neuronal density (per mm^3^)	Cross-sectional area (µm^2^)	Regional volume (mm^3^)
**Caudate-Putamen**				
Wild-type 4.5 months	2,147,747±45,204	126,548±1,027	96.17±0.91	17.19±0.84
Heterozygous 4.5 months	2,031,760±118,776 (−5.40%)	127,960±8,876 (+1.12%)	94.52±2.36 (−1.72%)	17.16±0.84 (−0.17%)
Homozygous 4.5 months	1,861,043±73,248 (−13.35%)*	111,929±6,068 (−11.55%)	93.79±1.44 (−2.47%)	15.31±0.52 (−10.94%)*
Wild-type 10 months	1,955,485±89,092	111,524±6,092	97.35±1.51	18.67±0.77
Heterozygous 10 months	1,908,482±59,861 (−2.40%)	108,611±4,377 (−2.61%)	93.65±1.30 (−3.8%)	17.70±0.50 (−5.20%)
Homozygous 10 months	1,658,100±39,419 (−15.21%)*	98,277±3,766 (−11.88%)	97.00±1.37 (−0.36%)	15.85±0.52 (−15.10%)*
**Nucl. Accumbens**				
Wild-type 4.5 months	446,709±17,531	118,866±4,731	89.10±1.10	3.77±0.14
Heterozygous 4.5 months	431,262±30,403 (−3.46%)	128,218±8,763 (+7.87%)	89.83±1.36 (+0.84%)	3.38±0.15 (−10.34%)
Homozygous 4.5 months	374,506±19,983 (−16.16%)*	123,233±6,462 (+3.67%)	89.90±1.28 (+0.91%)	3.05±0.10 (−19.10%)*
				
Wild-type 10 months	351,003±8,445	94,308±3,563	94.81±0.83	3.75±0.16
Heterozygous 10 months	356,054±11,075 (+1.44%)	93,835±3,755 (−0.50%)	94.03±1.51 (−0.82%)	3.80±0.06 (+1.33%)
Homozygous 10 months	311,319±7,077 (−11.31%)	105,589±5,589 (+11.96%)	92.75±1.92 (−2.17%)	2.99±0.18 (−20.27%)*
**Striatum (CPu+NA)**				
Wild-type 4.5 months	2,594,456±56,760	123,921±3,005	92.51±0.90	20.96±0.42
Heterozygous 4.5 months	2,463,021±126,696 (−5.07%)	120,319±7,066 (−2.91%)	92.06±1.84 (−0.49%)	20.54±0.45 (−2.00%)
Homozygous 4.5 months	2,235,550±86,136 (−13.83%)*	121,949±5,172 (−1.59%)	91.81±1.24 (−0.76%)	18.36±0.25 (−12.40%)*
Wild-type 10 months	2,306,489±92,700	102,789±3,102	96.12±1.14	22.41±0.37
Heterozygous 10 months	2,264,537±61,960 (−1.82%)	105,414±3,519 (+2.55%)	93.82±1.14 (−2.39%)	21.51±0.21 (−4.02%)
Homozygous 10 months	1,969,419±43,469 (−14.61%)*	104,647±2,660 (+1.81%)	94.91±1.45 (−1.26%)	18.84±0.37 (−15.93%)*

Estimated neuronal number, neuronal density, cross-sectional area and regional volume in caudate putamen, nucleus accumbens and striatum in 4.5 and 10 months old zQ175 and WT mice based on stereologic analysis. Percent change is calculated for each region by the following formula: % change = (experimental−wild-type)/wild-type * 100. Values that are significantly different from age-matched wild-type mice are indicated with *.

#### Brain Volume

The CPu volume increased with age in wild-type mice (F(1,30) = 13.9, p<0.001). No age-related changes in CPu volume were observed in heterozygous or homozygous mice. No significant age-related difference was detected in the NA in wild-type mice. However, in heterozygous mice, the NA was larger at 10 months than at 4.5 months (F(1,30) = 4.7, p = 0.038). Similarly, the volume of both CPu and NA were significantly reduced at both 4.5 and 10 months old in homozygous animals, when compared to wild-type and heterozygous animals (CPu −10.94% and −15.10% at 4.5 and 10 months of age, respectively; and NA −19.10 and −20.27% at 4.5 and 10 months of age, respectively). Total striatal volume of homozygous mice was significantly changed (F(5,30) = 19.2, p<0.001), when compared to age-matched wild-type and heterozygous mice (Table 2). MRI volumetry revealed a similar reduced volume in striatum at both 4 and 12 months of age when compared to wild-type animals (Fig. 5). At 10 months, striatal volume was increased in all groups compared to their genotype matched younger cohort, however only wild-type mice showed a statistically significant increase in striatal volume with age.

#### Neuronal number and cross-sectional area

Stereological analysis of NeuN-immunoreactive cells showed that neuronal number was highest in 4.5 month old wild-type animals (wild-type>heterozygous>homozygous) in all regions examined. In the CPu, homozygous mice had significantly fewer neurons when compared to wild-type animals at both 4.5 and 10 months of age (−13.35 and −15.21%, respectively; F(5,30) = 4.8, p = 0.003). Homozygous mice also had significantly fewer neurons in the NA at 4.5 months (F(5,30) = 8.5, p<0.001) compared to age-matched wild-type mice. No significant differences were seen in CPu or NA between heterozygous mice and wild-type at either age. At 10 months of age, NA neuronal number was decreased in all genotypes (wild-type, heterozygous and homozygous mice) when compared to genotype-matched young (4.5 month) mice (F(1,30) = 6.4–14.7, p = 0.017-0.001). While wild-type mice showed the greatest decrease with age, this is likely due in part to the early loss of neurons in transgenic animals by 4.5 months. When the CPu and NA are considered together as the striatum, total striatal neuron number was significantly reduced in both 10 month-old homozygous and wild-type mice when compared to genotype-matched 4.5 month-old mice (F(1,30) = 5.2–6.1, p = 0.02–0.03; Table 2). In addition, homozygous mice demonstrated significantly fewer neurons than wild-type age-matched controls at 4.5 months (cf.2.24×10^6^ vs. 2.60×10^6^) and 10 months of age (1.97×10^6^ vs. 2.31×10^6^). Neuronal cross-sectional area was not significantly different within the striatum or the caudate putamen, (*F*(5,30) = 1.1–1.7; *p* = 0.16–0.37). While no change in cell size was observed in homozygous mice, a significant increase in cell size was observed in the nucleus accumbens for heterozygous and wild-type mice at 10 months as compared to genotype-matched 4.5 months groups (*F*(5,30) = 2.8; *p* = 0.03).

#### Neuronal density

Because of concomitant changes in volume and cell number, neuronal density was reduced in wild-type and homozygous mice at 10 months when compared to genotype matched mice 4.5 months of age in CPu (F(1, 30) = 6.8–9.7; p = 0.004–0.01). The neuronal density in NA was also significantly reduced as function of age in all genotypes (F(1,30) = 4.7–17.8, p = 0.001). In the striatum, each group had also significantly reduced neuronal density at 10 months of age than in their younger cohorts (F(1,30) = 5.8–11.7, p = 0.009–0.022; Table 2). There were no differences in cell density observed due to genotype.

## Discussion

We have evaluated the onset, progression and magnitude of behavioral, electrophysiological, morphological and brain metabolic changes in the new zQ175 KI mouse model of HD. Body weight decrease is a commonly observed manifestation of progressive disease in many HD mouse models and also occurs in HD patients [34]. Decreased body weight manifests in zQ175 KI mice as a failure to gain weight comparable to a rate observed in WT mice, and is similar to that described in the accompanying manuscript [1]; those authors report that female homozygotes (from 7 weeks) and female heterozygotes (from 10 to 15 weeks) were lighter than WT females. It is conceivable that the behavioral tests at early ages (e.g., grip strength, Phenocube) described by those authors contributed to the slower body weight gain in both homozygote and heterozygote zQ175 mice, which could contribute to the differences in body weights between both cohorts. Our recent observations suggest that extensive testing and handling correlates with body weight differences between heterozygous zQ175 and WT mice as early as 30 weeks (unpublished observations).

Changes in voluntary and involuntary movement are apparent in HD; chorea, followed by bradykinesia and dystonia, are common. Changes in motor function have been reported in other HD mouse models; YAC 128 mice show open field hyperactivity at 3 months followed by decreased activity at 12 months [35]. The hyperactivity phase in HD mouse models, especially those with rapid disease progression, is often difficult to detect and is transient, converting rapidly to a hypokinetic stage [36], suggesting that the indirect and direct pathways in the striatum may be dysregulated. In both homo- and heterozygous zQ175 mice, hyperactivity was not observed in the open field, even at the earliest time point (2 months), but non-significant trends towards increased rearing activity in heterozygotes (2–4 months) and increased climbing activity in homozygotes (2 months) may indicate early hyperactivity. However, any hyperactivity phase measured by conventional open field or climbing measures, is not very robust and is transient in the zQ175 mice. Hypoactivity, as measured by decreased horizontal activity in the open field, was seen in homozygotes (from 2 months) and in heterozygotes (at 4 and 8 months). This hypolocomotor activity is similar to that described by Menalled et al. [1]. Together, these results show that both homo- and heterozygous zQ175 mice exhibit motor deficits early, at 2 and 4 months of age, respectively, which manifest prominently as a hypolocomotor phase.

Cognitive function was measured in a procedural two-choice swim test; this kind of procedural stimulus-response task presumably depends on intact striatal circuitry [18]. WT mice learned the task quickly, performing at close to 70% accuracy on test day 2 at 10 months. Heterozygous zQ175 mice performed almost as well as WT, whereas homozygotes were clearly impaired at 10 and 12 months. The striatum-dependent cognitive deficits of homozygotes at 10–12 months are not as advanced as those observed in symptomatic 9-week-old R6/2 mice (unpublished results). We re-tested zQ175mice at 12 months to see whether they had recall of the test session 2 months previously. Both WT and heterozygous mice seemed to have recall since they showed 60% accuracy on test day 1, increasing to 80–90% on day 2. In contrast, homozygotes performed equally poorly on both test sessions at 10 and 12 months suggesting no recall, underscoring their cognitive deficiency in tasks requiring intact striatal circuitry. In the accompanying manuscript [1] the homozygotes are significantly impaired in the same procedural task at 14 months. Such deficits in simple procedural learning have not been detected in other HD KI mouse models [9]. However, recent work in R6/1 mice has shown impaired neuronal function in the corticostriatal pathway, associated with deficits in procedural learning [37]. Interestingly, even though we show the corticostriatal neurotransmission of both homo- and heterozygotes is significantly impaired (more so in the former), only homozygotes display cognitive deficits in this task, perhaps indicating that the corticostriatal neurotransmission deficits in heterozygotes are not sufficient to impair the cognitive capacity required for this specific procedural task.

Medium spiny neurons (MSNs) are particularly vulnerable to m*HTT* insult and dramatic loss of this neuronal population in HD patients is a hallmark of the disease [7], [35], [38]. Other HD mouse models have shown that MSN neuronal and synaptic function becomes severely disrupted as the animals age [reviewed in 39], [40]–[42]. MSNs in symptomatic R6/2 mice show pronounced morphological abnormalities, including dendritic shrinkage and spine loss, and display a marked increase in membrane resistance, depolarization of the resting membrane potential, and an increased intrinsic excitability in response to current injection [19], [20], [39], as well as impaired corticostriatal connectivity [25]–[31]. We therefore determined the electrophysiological phenotype of MSNs taken from zQ175 mice, and our data are completely consistent with these earlier findings. MSNs in both the heterozygotes and homozygotes are more excitable than WT counterparts, with heterozygotes showing a progressive phenotype between 3–4 months and 6–9 months. Corticostriatal transmission is severely attenuated in both heterozygotes and homozygotes from very early ages. Interrogation by miniature EPSC analysis would implicate whether a strong reduction in synapse number or a reduction in presynaptic glutamate release is responsible for this phenotype.

In pre-manifest and manifest HD patients, brain atrophy is one of the most consistent and early pathological changes [43]; striatal atrophy, namely in caudate and putamen, may reach up to 41 and 54% shrinkage in mild to moderate HD [7], [44] and measurable atrophy is evident 15–20 years before disease onset [38], [45], [46]. Striatal atrophy is also a hallmark of several HD mouse models [15], [35], [47], [48]. Compared to WT, the whole brain volume of homozygous zQ175 mice decreases by up to 11% at 12 months, striatal atrophy reaches 21% loss at 8 months, and a 15% decrease in cortical volume is evident by 12 months. Consistent with the MRI, stereology revealed significant decrease in striatal regional volume at 4.5 months in homozygotes, remaining constant at 10 months. However, our recent unpublished MRI data show that decreased brain regional volumes (5% in whole brain, 9% in both striatum and cortex) are evident as early as 6 weeks in homozygotes, indicating very early pathology and possibly developmental defects due to m*Htt* expression from both alleles in zQ175 mice. In contrast, we saw no early MRI changes in brain regional volumes in heterozygotes, followed by a slowly progressing decline over a 12-month period; consistent with this, stereological changes in brain regional volume were not evident at early ages in heterozygotes. However, in contrast to MRI, stereology indicated only trends toward decreased brain regional volumes. This difference may be due to smaller N values and larger variation, possibly due to use of both sexes in the stereology studies.

Given that the CAG repeat in the zQ175 mice is in the high juvenile HD range, a comparison of the time course and magnitude of changes to that seen in early stage juvenile HD may be instructive. Furthermore, in regard to brain regional changes, the heterozygous zQ175 mice may be more suitable in studies evaluating this outcome measure as they do not show the very early (possibly developmental) changes reported in the homozygotes.

We report here a decrease in neuronal number, measured by stereology, as early as 4.5 months in homozygotes, remaining constant at 10 months. At 4.5 months the striatal neuronal number was decreased 5% in heterozygotes and 14% in homozygotes compared to WT. Striatal neuronal loss has been reported in some transgenic HD mouse models; N171-82Q mice expressing 82 CAG repeats have as much as 39% fewer NeuN-positive neurons in striatum compared to WT [49]. A MRS study of the brain metabolism in healthy volunteers and participants with pre-symptomatic or early stage HD found that the striatal concentrations of NAA were already lower at the pre-HD stage, correlating with impaired cognitive function [50]. Lower concentrations of striatal NAA and creatine have also been reported in HD patients [51], [52]. Here we report decreased levels of NAA in homozygotes from 4 months, at motor symptom onset; there could be a connection between the lower striatal NAA and motor and cognitive performance since decreased NAA may indicate neuronal dysfunction and death [53]. Interestingly, compared to WT the creatine levels in both heterozygotes and homozygotes were increased, in contrast to humans [52]. Concentrations of striatal myo-inositol have been reported to increase in early HD [54], and we observed increased myo-inositol levels in the heterozygotes as late as 12 months. Metabolic profiles from zQ175 brain tissue at 12 months closely resemble those in the well characterized R6/2 mouse [55]; increased levels of glutamine, myo-inositol, taurine, and creatine+phosphocreatine and decreased NAA in both lines.

MRS data are normally given either as concentration ratio relative to total creatine or as absolute concentration (in reference to water signal acquired from same volume of interest). Increased creatine levels are common in HD mouse models while clinical MR studies indicate decreased striatal creatine levels. Although the direction of creatine change is intriguing in an HD context, creatine ratios should be avoided since changes in concentration ratio of a metabolite may be a result of either true change or from a change in creatine levels. While brain water homeostasis is very efficient, increased metabolite levels reported here raise the possibility that there is a significant contribution from altered NMR visible water content in both hetero- and homozygotes as a function of age, HD pathology, or combination of the two. Interestingly, the metabolic changes in HD and hypernatremia are similar [55]. Mild chronic hypernatremia could explain the dehydration of the brain. Both taurine and myo-inositols are osmolites and their increased levels may also be influenced by the altered water environment in zQ175 striatum.

In summary, the behavioral, electrophysiological, histological, and metabolic imaging measurements reported here indicate that the progressive phenotypes in the zQ175 mice up to 12 months of age recapitulate some aspects of HD. The robustness of the phenotypes, their dosage sensitivity, and their progressive nature makes this new mouse line a promising model for pharmacological or genetic testing to identify mechanisms that can modulate HD progression.

## Supporting Information

Table S1 **MRI volmetry power analysis in zQ175 and WT mice.** Summary of the sample size needed to detect a 50% effect in the MRI volumetry for whole brain, striatum and cortex, and in the concentrations of selected MRS striatal metabolites with an alpha of 0.05 and a power of 0.8 for the heterozygote (HET) and homozygote (HOMO) mice at 12 months of age. N/A, not applicable.
(DOC)
