# Supplementary material for: Characterization of Neurophysiological and Behavioral Changes, MRI Brain Volumetry and 1H MRS in zQ175 Knock-In Mouse Model of Huntington's Disease
Source: PLoS One. 2012 Dec 20;7(12):e50717. doi: 10.1371/journal.pone.0050717 (PMC3527436; doi:10.1371/journal.pone.0050717)
Supplement: Table S1 — MRI volmetry power analysis in zQ175 and WT mice. Summary of the sample size needed to detect a 50% effect in the MRI volumetry for whole brain, striatum and cortex, and in the concentrations of selected MRS striatal metabolites with an alpha of 0.05 and a power of 0.8 for the heterozygote (HET) and homozygote (HOMO) mice at 12 months of age. N/A, not applicable. (DOC) [file pone.0050717.s001.doc]

Supplemental Table 1

|  | zQ175 WT vs. zQ175 HET  Sample size | zQ175 WT vs. zQ175 HOMO  Sample size |
| --- | --- | --- |
|
| Whole Brain Volume | 24 | 12 |
| Striatum Volume | 26 | 9 |
| Cortex Volume | 44 | 8 |
| GLN | 78 | 14 |
| NAA | N/A | 26 |
| CR+PCR | 21 | 29 |

Supplemental Table 1. Summary of the sample size needed to detect a 50% effect in the MRI volumetry for whole brain, striatum and cortex, and in the concentrations of selected MRS striatal metabolites with an alpha of 0.05 and a power of 0.8 for the heterozygote (HET) and homozygote (HOMO) mice at 12 months of age. N/A, not applicable.
